# Supplementary material for: Tailoring superstructure units for improved oxygen redox activity in Li-rich layered oxide battery’s positive electrodes
Source: Nat Commun. 2024 Nov 18;15:9981. doi: 10.1038/s41467-024-54312-z (PMC11573992; doi:10.1038/s41467-024-54312-z)
Supplement: Supplementary file 1 — Supplementary Information [file 41467_2024_54312_MOESM1_ESM.pdf]

Supplementary Information

Tailoring superstructure units for improved oxygen redox activity in Li-rich layered oxide battery's positive electrodes

Hao Liu[1], Weibo Hua[1,2]*, Sylvia Kunz[3], Matteo Bianchini[3], Hang Li[1,4], Jiali Peng[1], Jing Lin[5], Oleksandr Dolotko[1], Thomas Bergfeldt[1], Kai Wang[5,6], Christian Kübel[5,6,7,8], Peter Nagel[8,9], Stefan Schuppler[8,9], Michael Merz[8,9], Bixian Ying[10], Karin Kleiner[10], Stefan Mangold[11], Deniz Wong[12], Volodymyr Baran[13], Michael Knapp[1], Helmut Ehrenberg[1], and Sylvio Indris[1,14]*

[1]Institute for Applied Materials (IAM), Karlsruhe Institute of Technology (KIT), Hermann-von-Helmholtz-Platz 1, 76344 Eggenstein-Leopoldshafen, Germany.

[2]School of Chemical Engineering and Technology, Xi'an Jiaotong University, No.28, West Xianning Road, Xi'an, Shaanxi 710049, China.

[3]University of Bayreuth, Bavarian Center for Battery Technology (BayBatt), Universitätsstraße 30, 95447 Bayreuth, Germany.

[4]School of Advanced Materials, Peking University, Shenzhen Graduate School, Shenzhen 518055, China.

[5]Institute of Nanotechnology (INT), Karlsruhe Institute of Technology (KIT), Hermann-von-Helmholtz-Platz 1, 76344 Eggenstein-Leopoldshafen, Germany.

[6]Department of Materials and Earth Sciences, Technical University of Darmstadt, 64287 Darmstadt, Germany.

[7]Helmholtz-Institute Ulm for Electrochemical Energy Storage (HIU), Karlsruhe Institute of Technology (KIT), Helmholtzstraße 11, 89081 Ulm, Germany.

[8]Karlsruhe Nano Micro Facility, Karlsruhe Institute of Technology (KIT), Kaiserstraße 12, 76131 Karlsruhe, Germany.

[9]Institute for Quantum Materials and Technologies, Karlsruhe Institute of Technology (KIT), Kaiserstraße 12, 76131 Karlsruhe, Germany.

[10]Münster Electrochemical Energy Technology (MEET), University of Münster (WWU), 48149 Münster, Germany.

[11]Institute for Photon Science and Synchrotron Radiation (IPS), Karlsruhe Institute of Technology (KIT), Hermann-von-Helmholtz-Platz 1, 76344 Eggenstein-Leopoldshafen, Germany.

[12]Dynamics and Transport in Quantum Materials, Helmholtz-Zentrum Berlin für Materialen und Energie, GmbH, Albert-Einstein-Strasse 15, 12489 Berlin, Germany.

[13]Deutsches Elektronen-Synchrotron (DESY), Notkestrasse 85, 22607 Hamburg, Germany.

[14]Applied Chemistry and Engineering Research Centre of Excellence (ACER CoE), Université Mohammed VI Polytechnique (UM6P), Lot 660, Hay Moulay Rachid, Ben Guerir, 43150, Morocco.

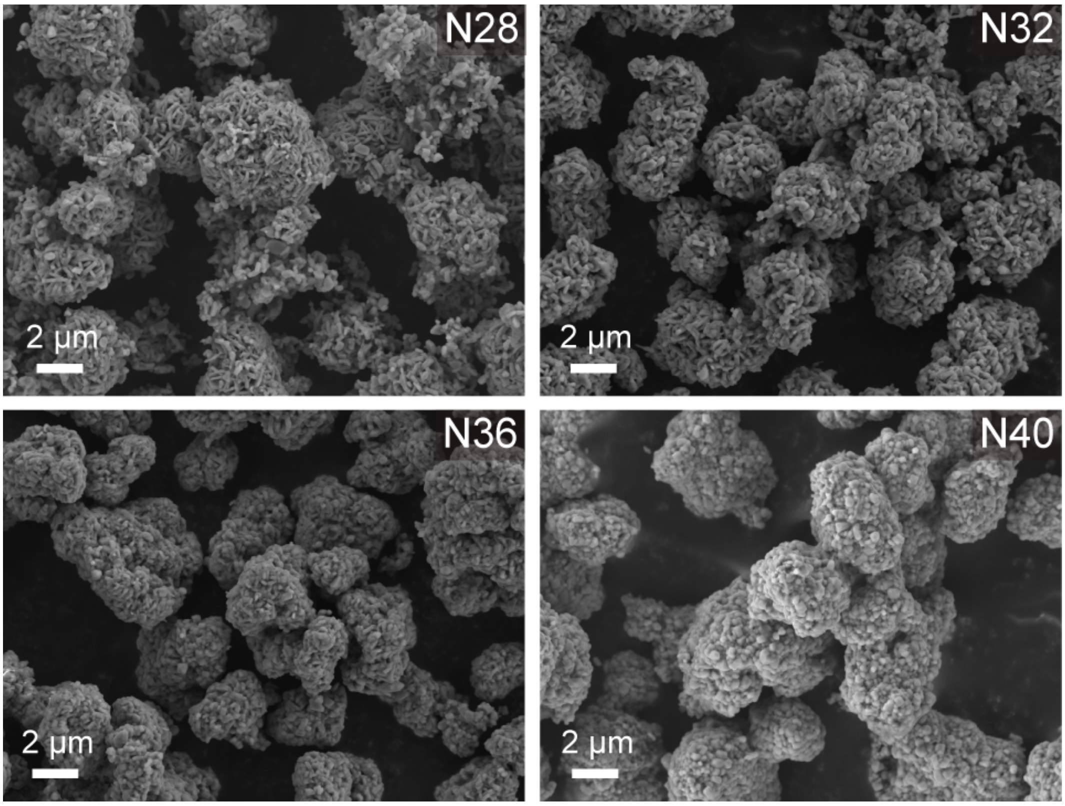

Supplementary Fig. 1 SEM images of N28, N32, N36, and N40 powders.

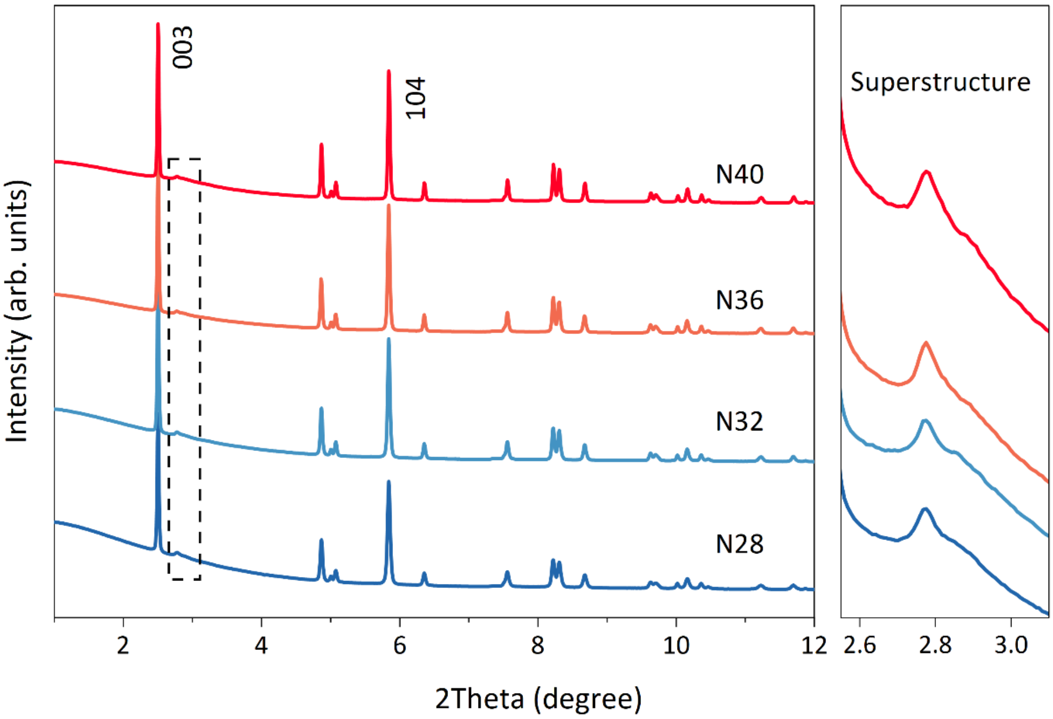

Supplementary Fig. 2 SXRD patterns for N28, N32, N36 and N40 powders. The wavelength at the beamline was 0.2073 Å

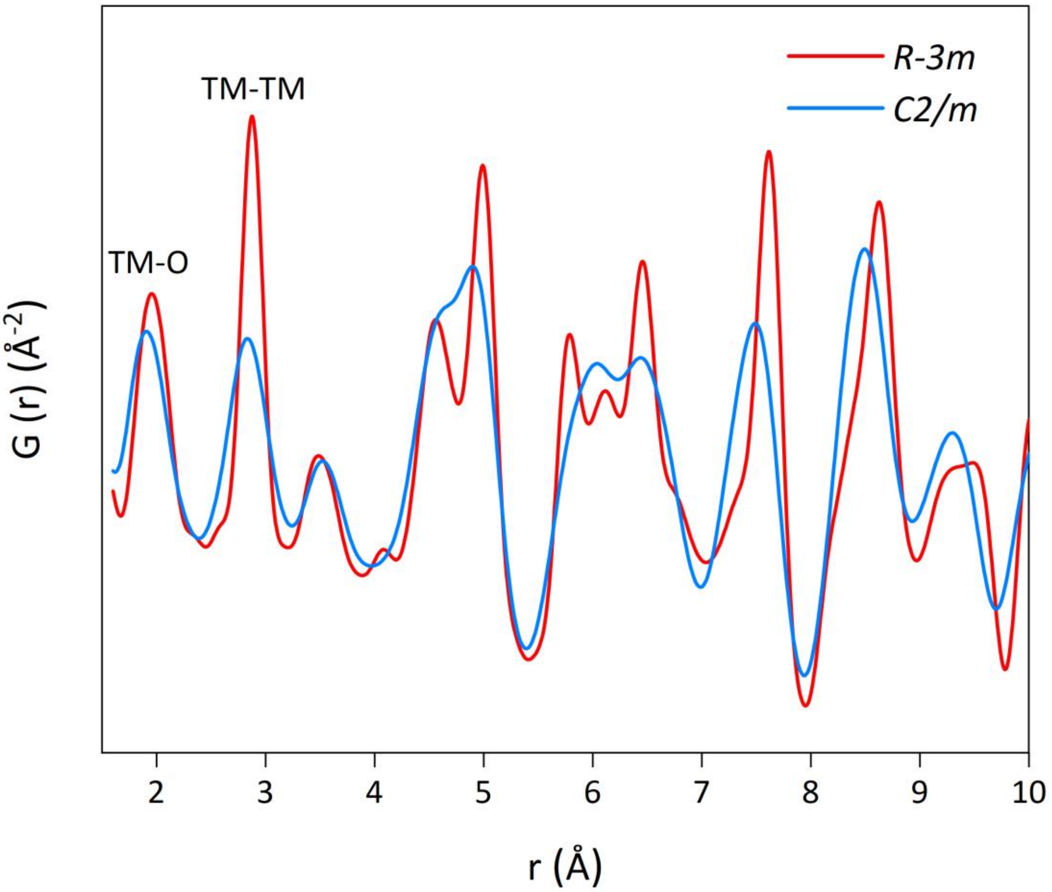

Supplementary Fig. 3 Calculated PDF patterns based on the *R-3m* and *C2/m* model.

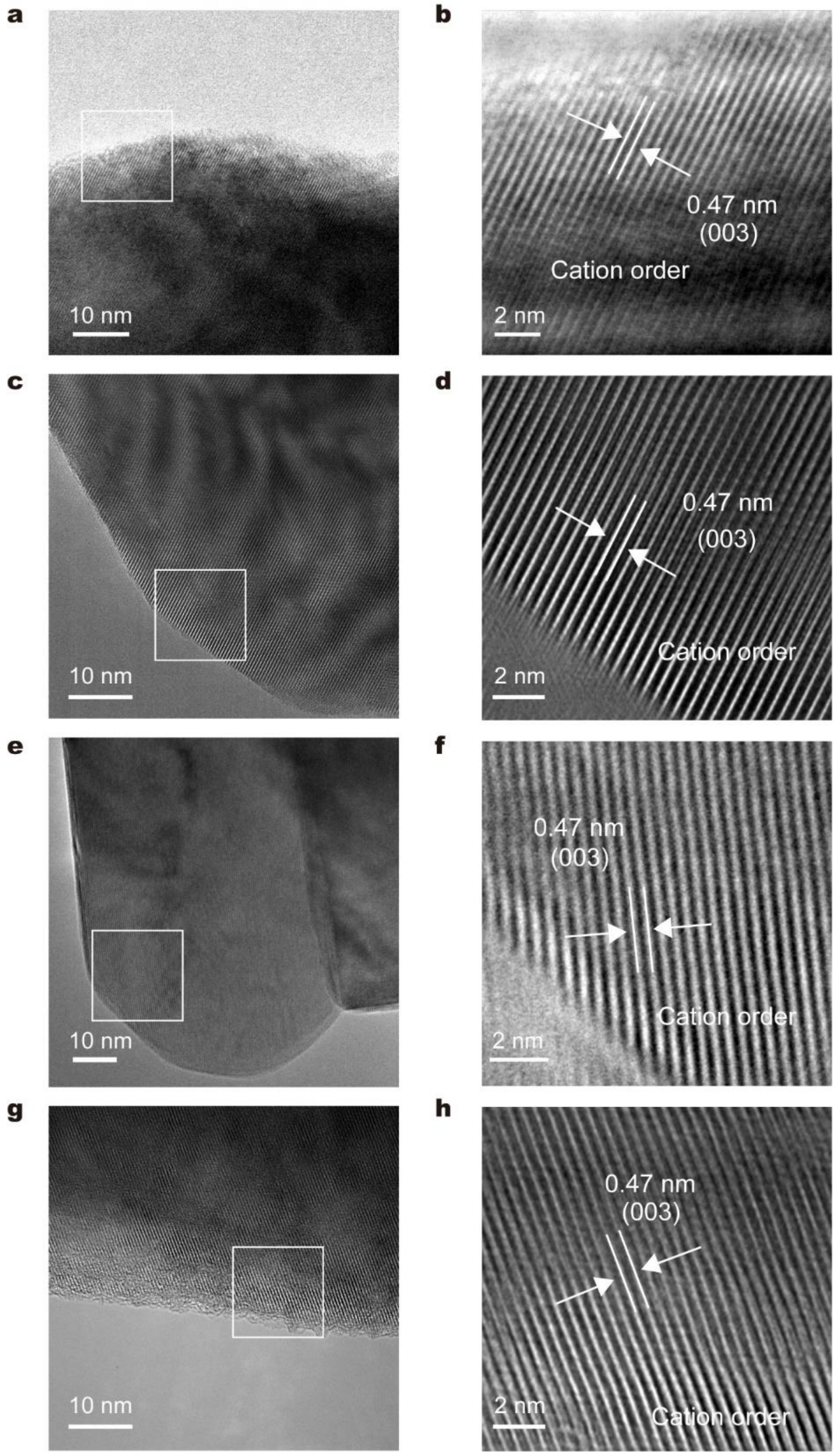

Supplementary Fig. 4 HRTEM images of N28 (**a-b**), N32 (**c-d**), N36 (**e-f**), and N40 (**g-h**) powders.

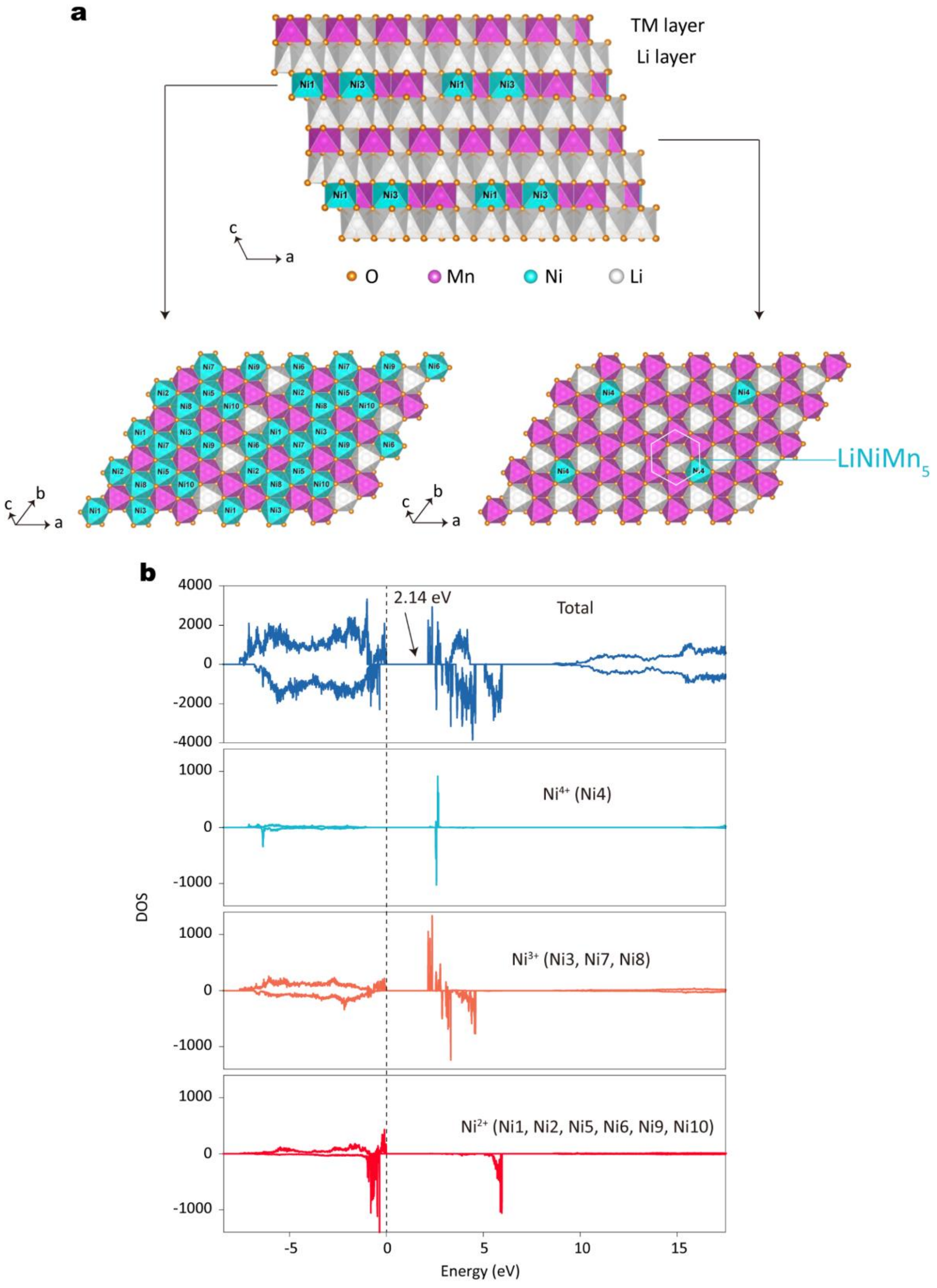

Supplementary Fig. 5 (**a**) Atomic structure model (top panel) and $LiNiMn_5$ honeycomb superstructure within TM layers (bottom panel). (**b**) The Total DOS and corresponding PDOS of Ni^{2+}, Ni^{3+}, and Ni^{4+} from the DFT calculation. The Fermi level is set to 0 eV and shown with a dashed line.

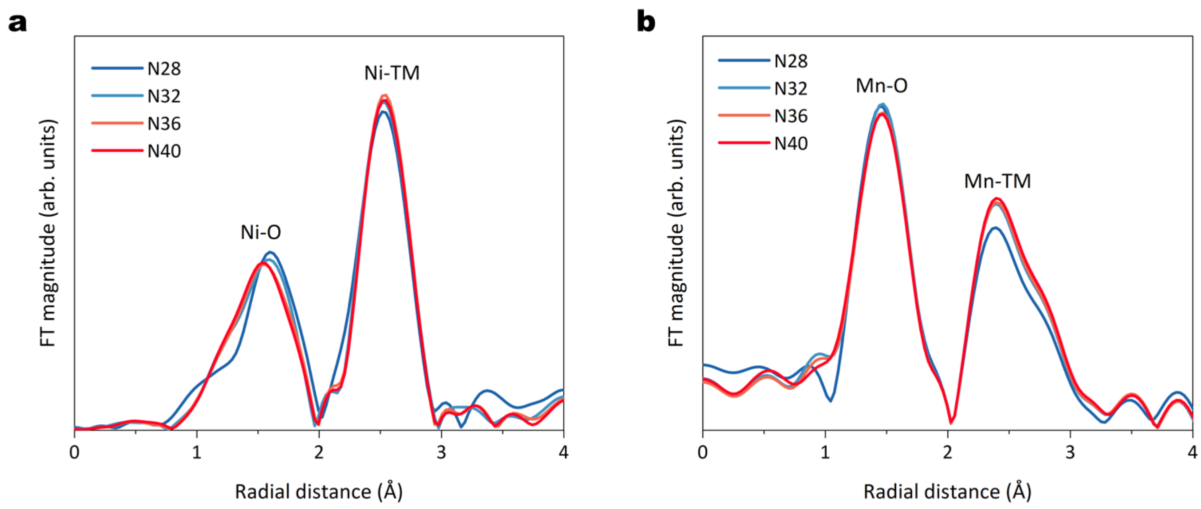

Supplementary Fig. 6 FT-EXAFS of transition metals Ni-K (**a**) and Mn-K (**b**) in N28, N32, N36 and N40 electrodes in the pristine state.

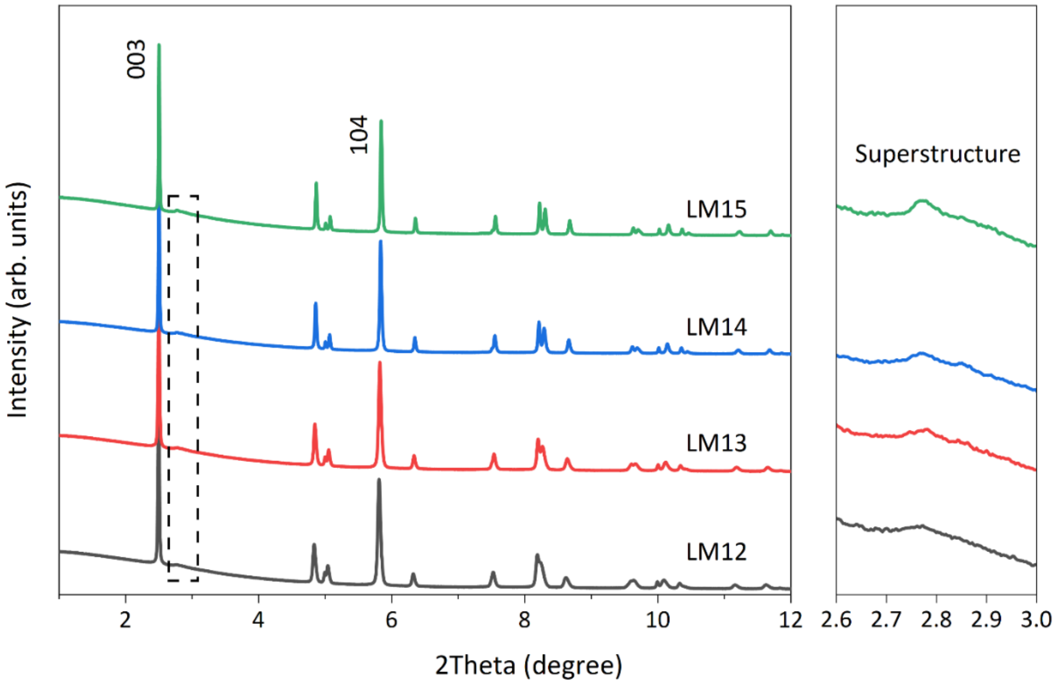

Supplementary Fig. 7 SXRD patterns for LM12, LM13, LM14 and LM15 powders. The wavelength at the beamline was 0.2073 Å.

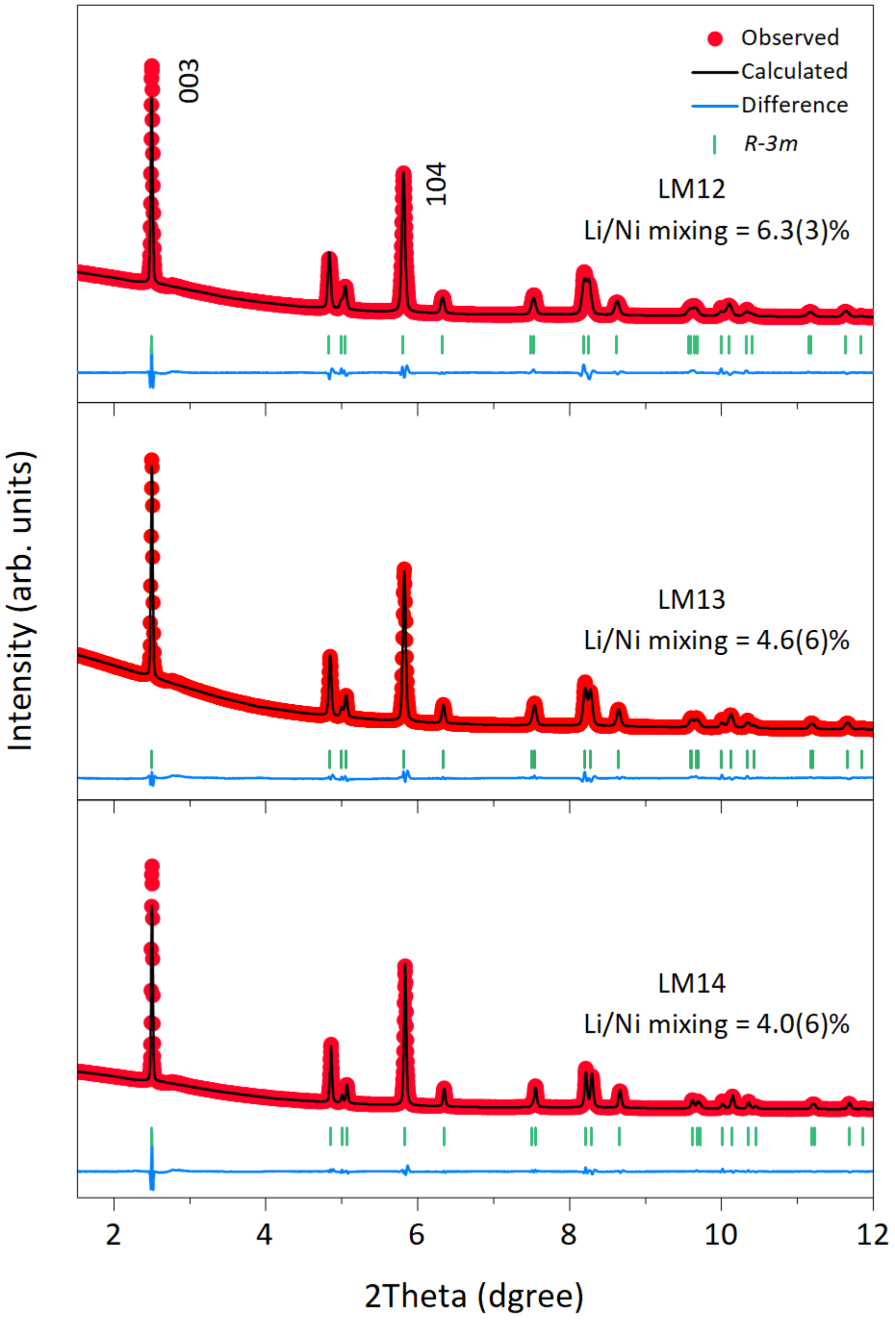

Supplementary Fig. 8 SXRD patterns and corresponding refinement results for LM12, LM13, and LM14 powders. The wavelength at the beamline was 0.2073 Å.

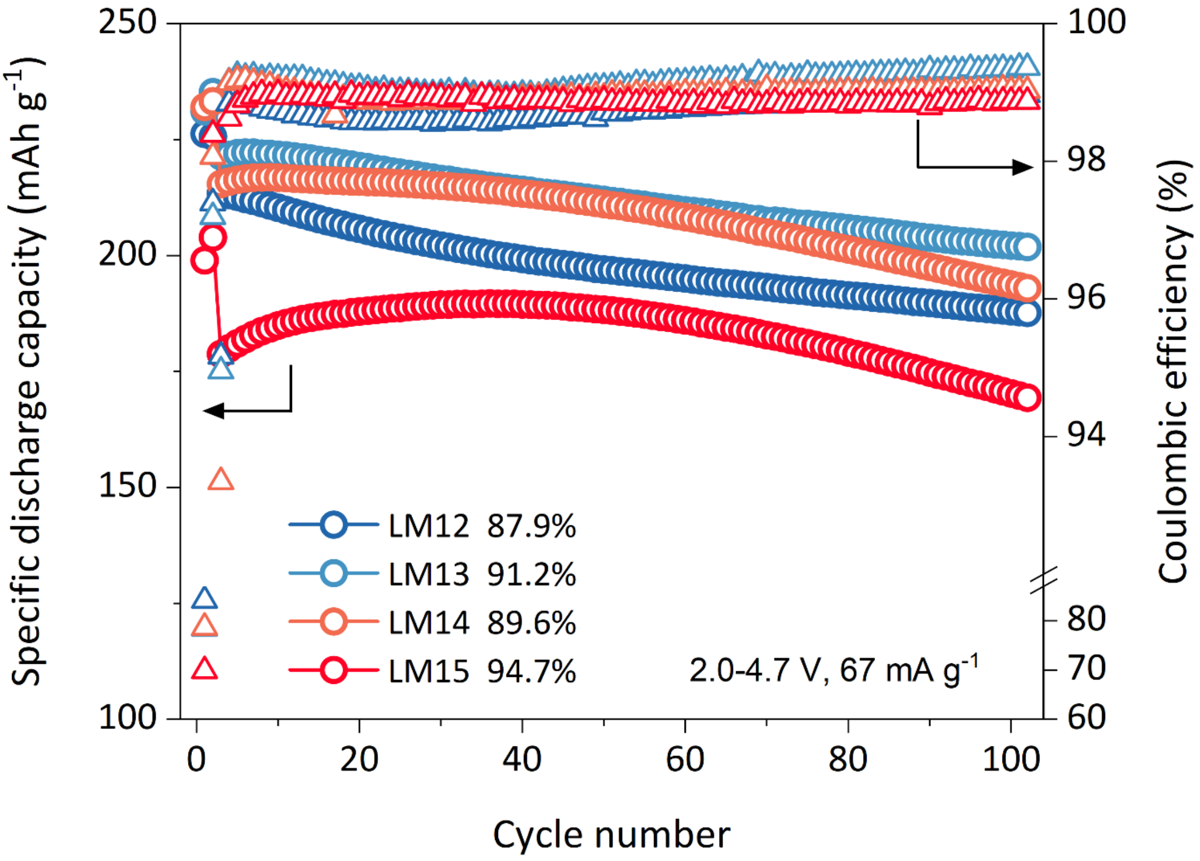

Supplementary Fig. 9 Cycling performance of Li metal coin cell with the LM12, LM13, LM14, and LM15-based positive electrodes at 67 mA g^{-1} (formation at 20 mA g^{-1} for two cycles) in the voltage range of 2.0-4.7 V at 25°C.

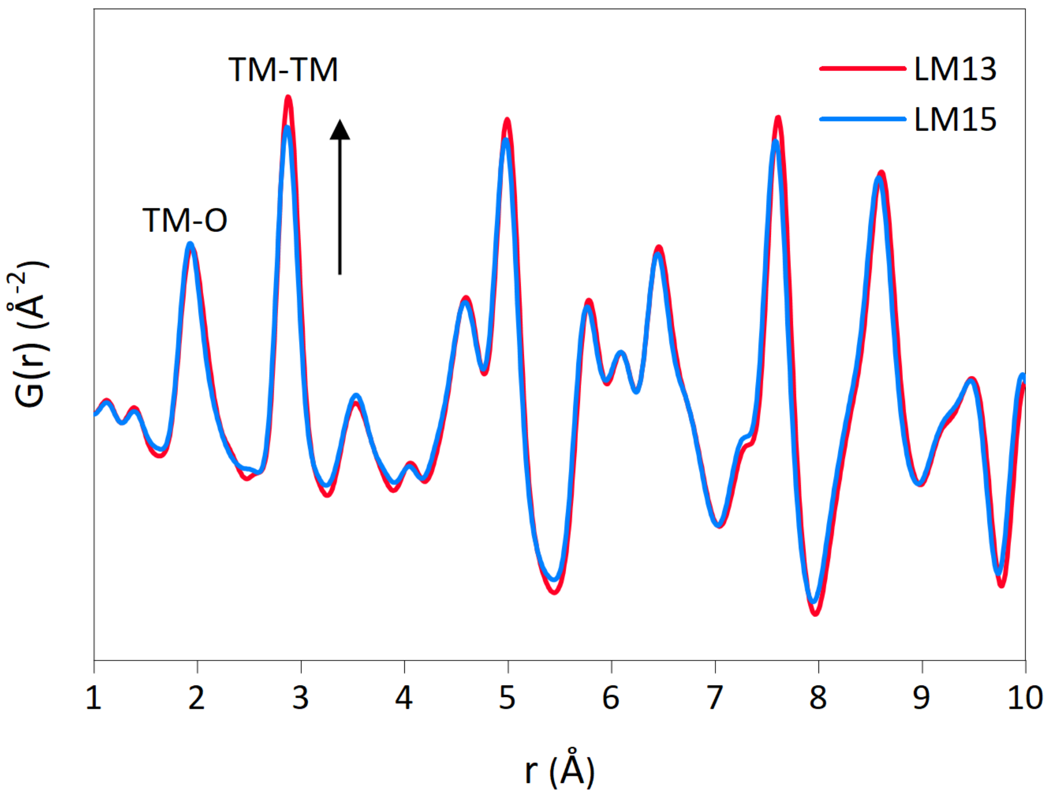

Supplementary Fig. 10 PDF patterns of LM15 and LM13 powders.

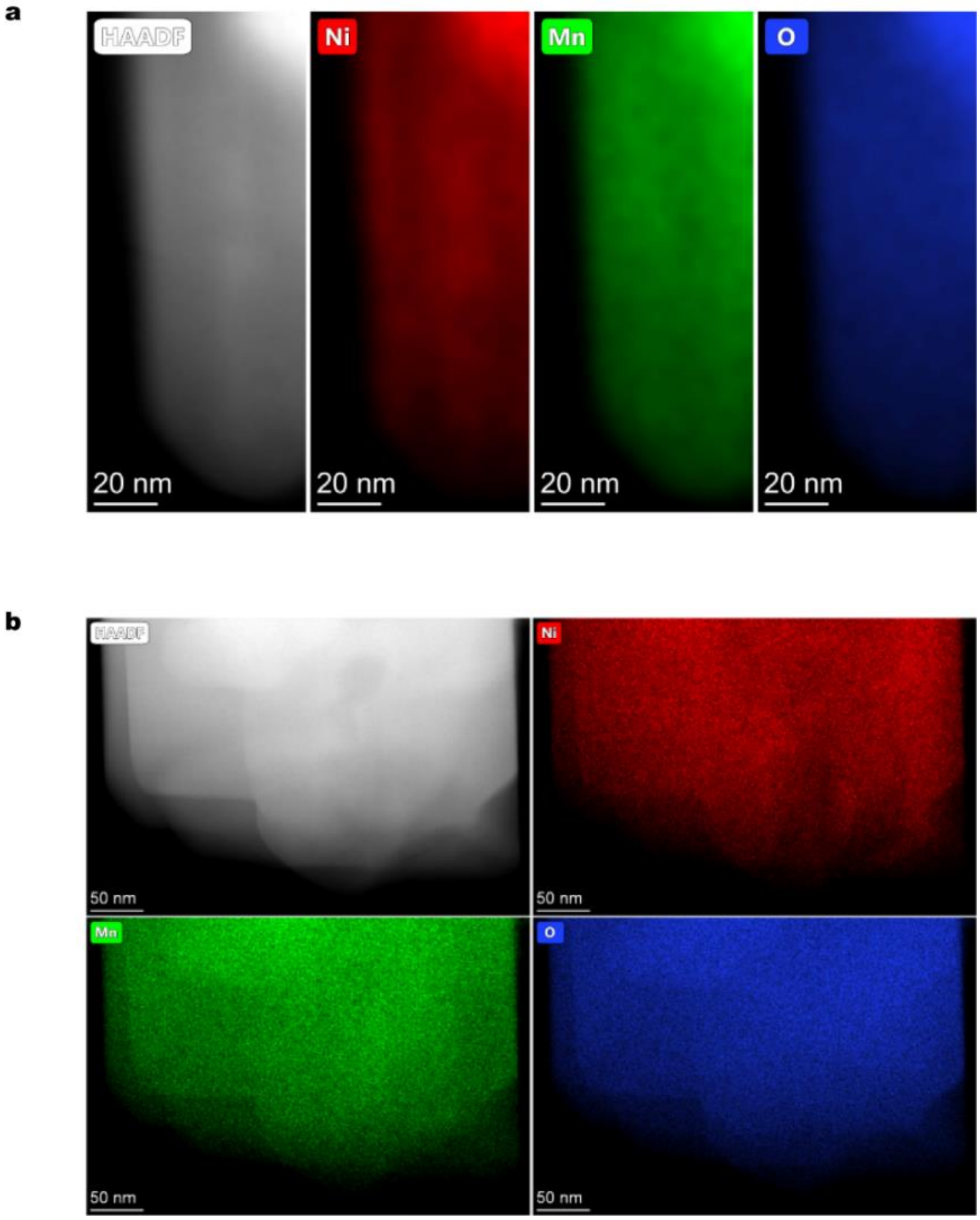

Supplementary Fig. 11 TEM-EDS mapping images of (**a**) LM15 and (**b**) LM13 powders.

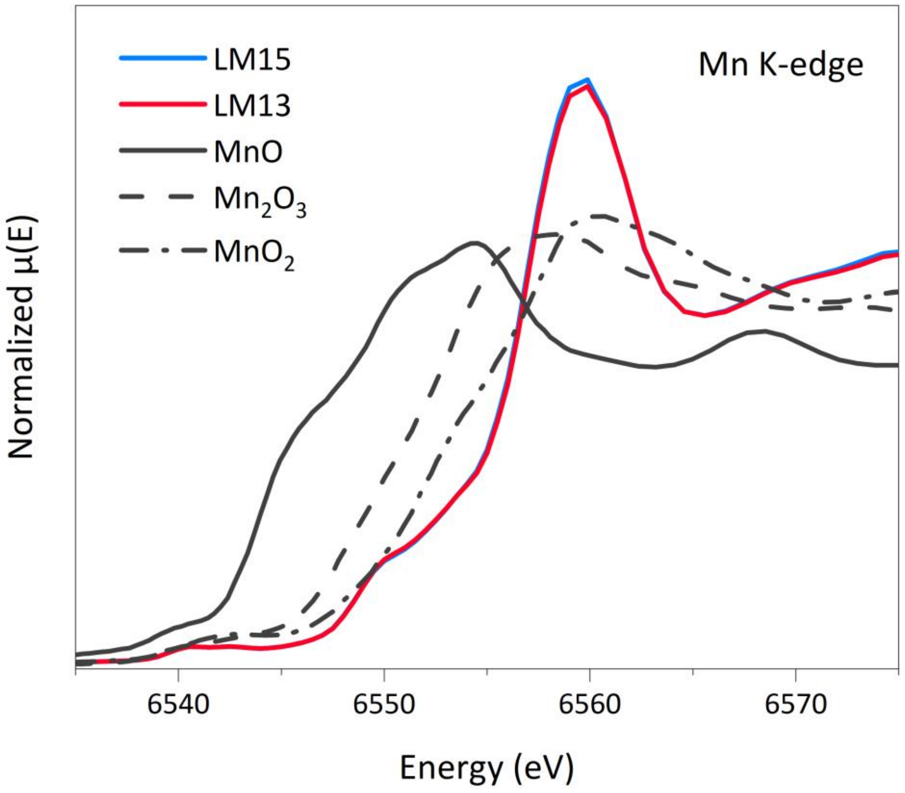

Supplementary Fig. 12 Normalized XAS Mn K-edge XANES of LM13 and LM15 electrodes in the pristine state. The XAS data are collected in transmission mode

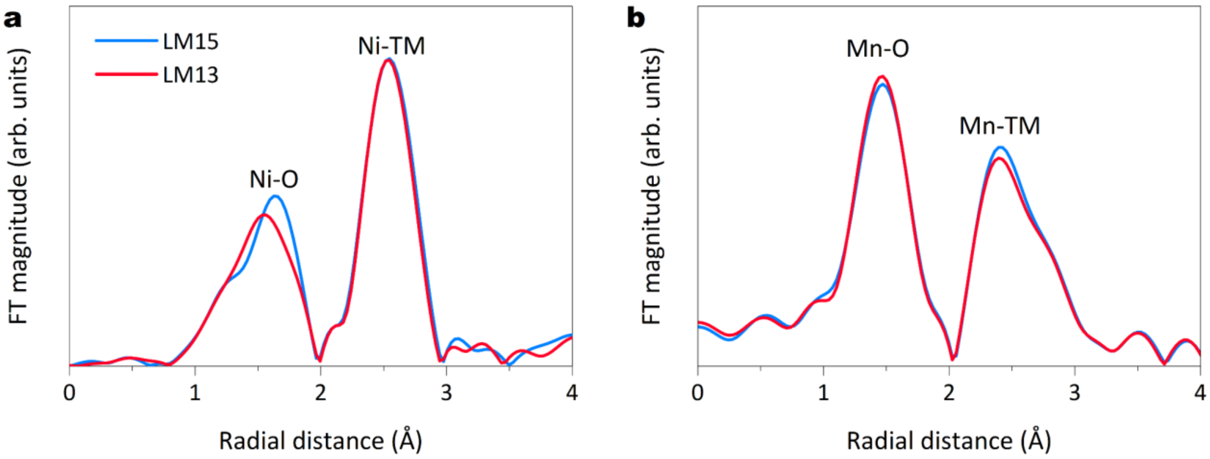

Supplementary Fig. 13 FT-EXAFS of transition metals Ni-K (**a**) and Mn-K (**b**) of LM13 and LM15 electrodes in the pristine state.

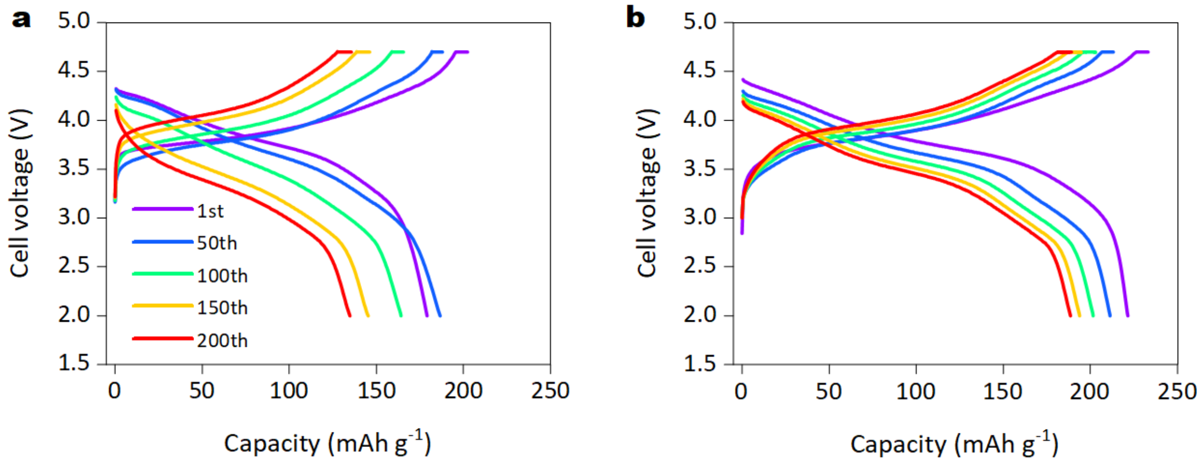

Supplementary Fig. 14 Charge/discharge curves (e.g. 1st, 50th, 100th, 150th, and 200th) for (**a**) Li||LM15 coin cell and (**b**) Li||LM13 coin cell.

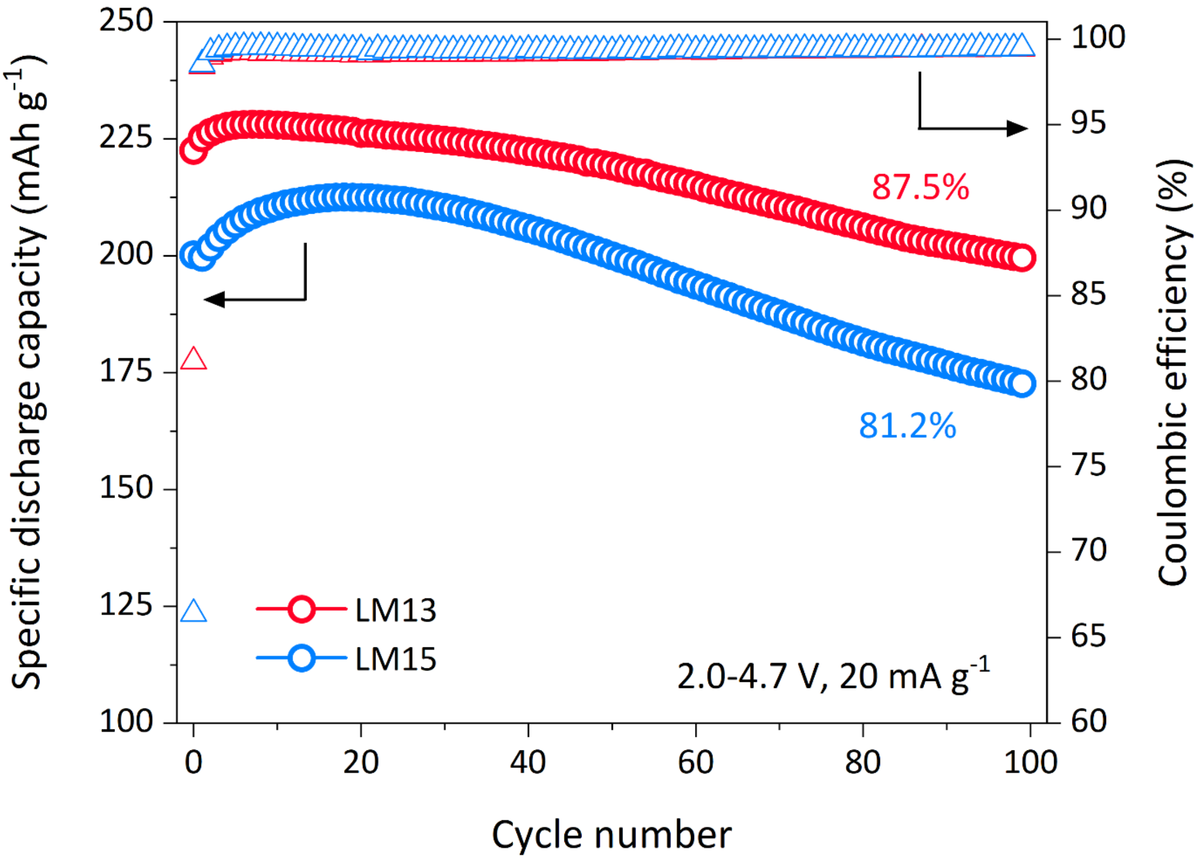

Supplementary Fig. 15 Cycling performance of Li||LM13 coin cell and Li||LM15 coin cell at 20 mA g^{-1} in the voltage range of 2.0-4.7 V at 25°C.

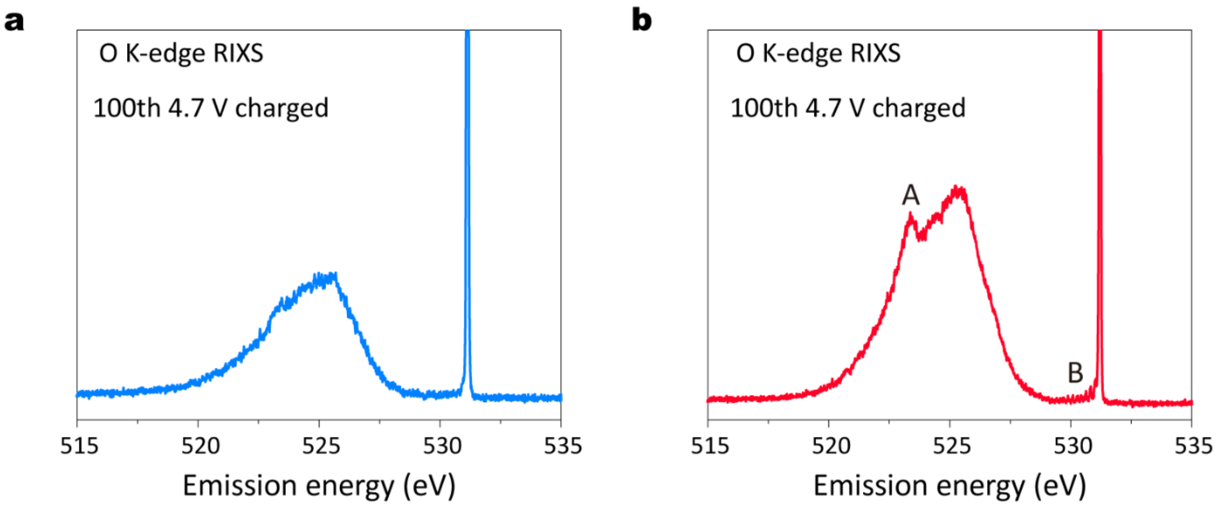

Supplementary Fig. 16 The O K-edge RIXS spectra collected at an excitation energy of 531 eV for (**a**) LM15 and (**b**) LM13 in the cycled electrodes at 20 mA g^{-1} after 100 cycles in the charged state at 4.7 V.

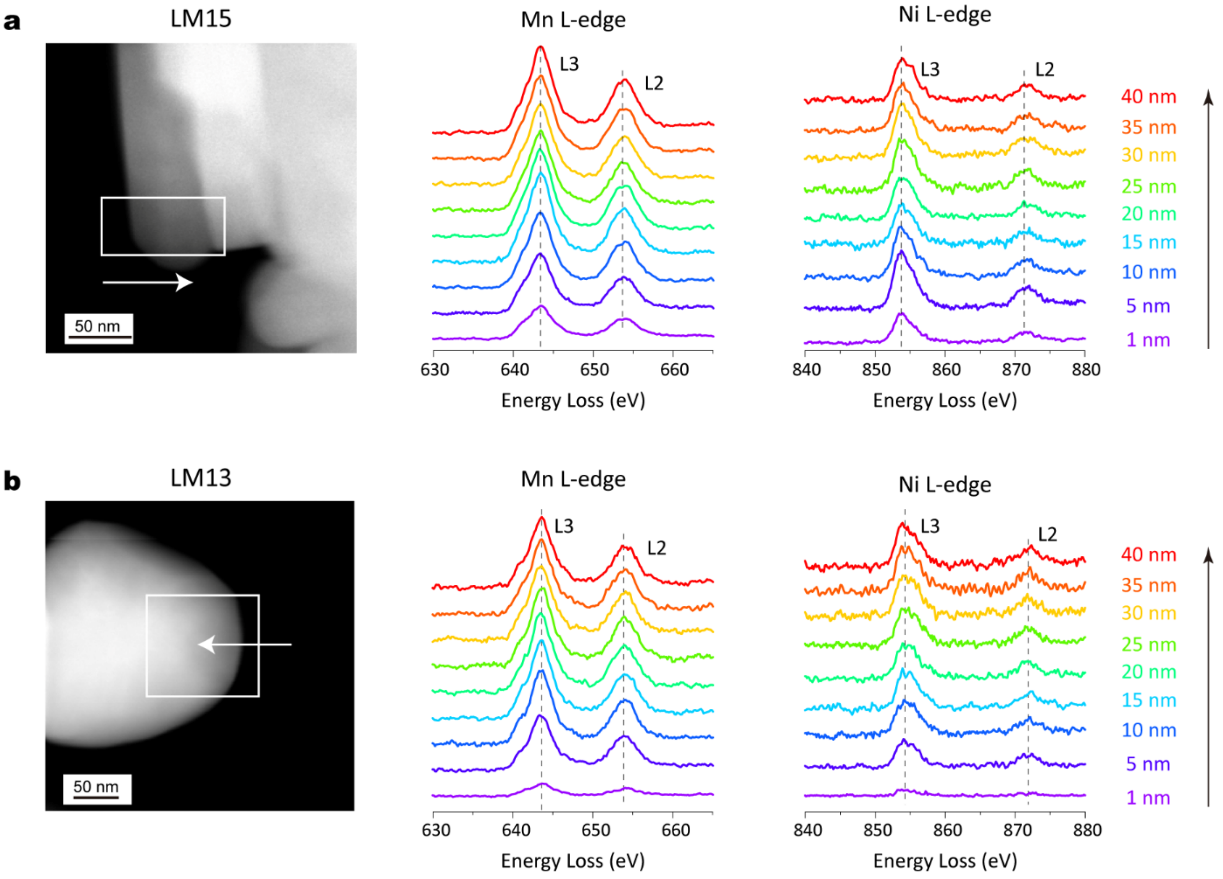

Supplementary Fig. 17 EELS line scans at particle surface (**a**) LM15 and (**b**) LM13 powders.

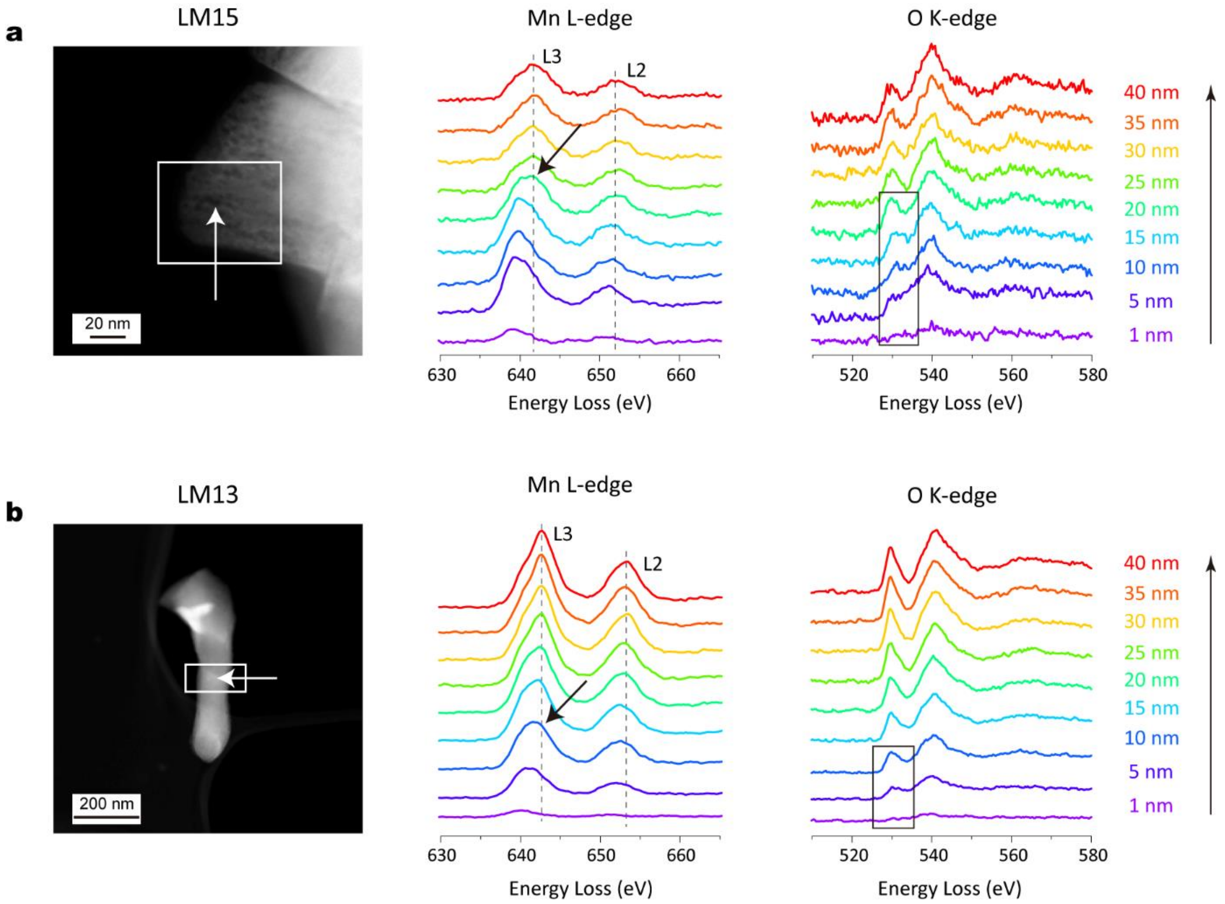

Supplementary Fig. 18 EELS line scans at particle surface (**a**) LM15 and (**b**) LM13 in the cycled electrodes at 67 mA g^{-1} after 200 cycles.

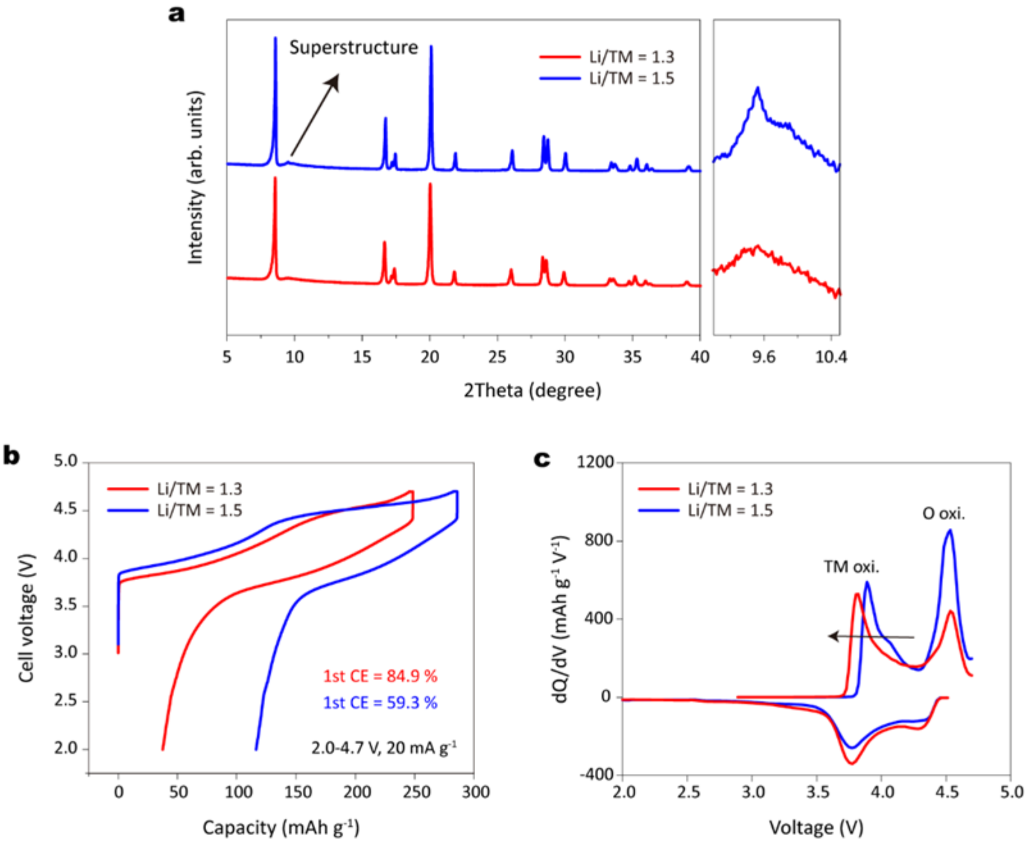

Supplementary Fig. 19 (**a**) XRD patterns for Li/TM = 1.3 and Li/TM = 1.5 powders. The patterns are acquired with a Mo source powder diffractometer in transmission geometry (wavelength of 0.7093 Å). (**b**) Charge and discharge curves and (**c**) corresponding dQ/dV curves of Li metal coin cell with the Li/TM = 1.3 and Li/TM = 1.5-based positive electrodes in the first cycle in the voltage range of 2.0-4.7 V at 20 mA g^{-1} at 25 °C.

Supplementary Table 1 Crystallographic data of N28, N32, N36, and N40 powders obtained from Rietveld refinement of SXRD pattern.

Atom	Site	x	y	z	Occ
Li1/Ni1	3b	0	0	0.5	0.976(4)/0.024(4)
Li2/Ni2/Mn1	3a	0	0	0	0.224(4)/0.255(4)/0.520
O1	6c	0	0	0.2576(6)	1

$Li_{1.20}Ni_{0.28}Mn_{0.52}O_2$ (N28), Space group: *R-3m*
a = *b* = 2.8598(1) Å, *c* = 14.2362(14) Å
R_{wp} = 1.78%, R_p = 1.14%

Atom	Site	x	y	z	Occ
Li1/Ni1	3b	0	0	0.5	0.977(4)/0.023(4)
Li2/Ni2/Mn1	3a	0	0	0	0.223(4)/0.297(4)/0.480
O1	6c	0	0	0.2577(5)	1

$Li_{1.20}Ni_{0.32}Mn_{0.48}O_2$ (N32), Space group: *R-3m*
a = *b* = 2.8602(1) Å, *c* = 14.2345(9) Å
R_{wp} = 2.00%, R_p = 1.25%

Atom	Site	x	y	z	Occ
Li1/Ni1	3b	0	0	0.5	0.978(4)/0.022(4)
Li2/Ni2/Mn1	3a	0	0	0	0.222(4)/0.338(4)/0.440
O1	6c	0	0	0.2568(4)	1

$Li_{1.20}Ni_{0.36}Mn_{0.44}O_2$ (N36), Space group: *R-3m*
a = *b* = 2.8611(1) Å, *c* = 14.2300(8) Å
R_{wp} = 2.44%, R_p = 1.51%

Atom	Site	x	y	z	Occ
Li1/Ni1	3b	0	0	0.5	0.982(4)/0.018(4)
Li2/Ni2/Mn1	3a	0	0	0	0.218(3)/0.382(4)/0.400
O1	6c	0	0	0.2577(5)	1

$Li_{1.20}Ni_{0.40}Mn_{0.40}O_2$ (N40), Space group: *R-3m*
a = *b* = 2.8593(1) Å, *c* = 14.2241(8) Å
R_{wp} = 2.11%, R_p = 1.36%

Supplementary Table 2 ^{6}Li NMR fitting results for N28, N32, N36, and N40 powders.

			N28			
Shift (ppm)	1.40	541.82	734.97	889.55	1330.58	1491.11
Area (%)	1.05	30.69	43.26	12.88	2.22	9.90

			N32			
Shift (ppm)	0.68	543.96	733.26	871.23	1316.23	1492.67
Area (%)	0.98	34.89	33.84	19.97	3.82	6.49

			N36			
Shift (ppm)	0.61	544.25	733.92	868.81	1306.3	1487.56
Area (%)	0.90	44.98	29.18	17.18	4.18	3.57

			N40			
Shift (ppm)	0.48	572.94	745.80	804.65	1304.38	1491.65
Area (%)	1.03	50.64	4.36	37.04	4.26	2.67

Supplementary Table 3 ICP–OES results for LM12, LM13, LM14, and LM15 powders.

Sample	Li	Ni	Mn	Li/TM
LM12	0.960	0.360	0.440	1.200
LM13	1.028	0.360	0.440	1.285
LM14	1.105	0.360	0.440	1.381
LM15	1.161	0.361	0.439	1.451

Supplementary Table 4 Crystallographic data of LM12, LM13, and LM14 powders obtained from Rietveld refinement of SXRD pattern.

Atom	Site	x	y	z	Occ
Li1/Ni1	3b	0	0	0.5	0.937(3)/0.063(3)
Li2/Ni2/Mn1	3a	0	0	0	0.153(3)/0.348(3)/0.500
O1	6c	0	0	0.2576(6)	1

$Li_{1.09}Ni_{0.41}Mn_{0.50}O_2$ (LM12), Space group: *R-3m*
a = *b* = 2.8787(1) Å, *c* = 14.2659(15) Å
R_{wp} = 4.02%, R_p = 2.11%

Atom	Site	x	y	z	Occ
Li1/Ni1	3b	0	0	0.5	0.954(6)/0.046(6)
Li2/Ni2/Mn1	3a	0	0	0	0.176(6)/0.344(6)/0.480
O1	6c	0	0	0.2574(11)	1

$Li_{1.13}Ni_{0.39}Mn_{0.48}O_2$ (LM13), Space group: *R-3m*
a = *b* = 2.8717(2) Å, *c* = 14.2519(24) Å
R_{wp} = 2.70%, R_p = 1.84%

Atom	Site	x	y	z	Occ
Li1/Ni1	3b	0	0	0.5	0.960(6)/0.040(6)
Li2/Ni2/Mn1	3a	0	0	0	0.210(6)/0.330(6)/0.460
O1	6c	0	0	0.2576(5)	1

$Li_{1.17}Ni_{0.37}Mn_{0.46}O_2$ (LM14), Space group: *R-3m*
a = *b* = 2.8649(1) Å, *c* = 14.2398(8) Å
R_{wp} = 3.75%, R_p = 1.94%

Supplementary Table 5 ^{6}Li NMR fitting results for LM13 powder.

			LM13			
Shift (ppm)	0.96	577.16	730.15	834.87	1332.84	1495.76
Area (%)	0.74	50.74	6.75	32.19	4.09	5.48

Supplementary Table 6 Atomic coordinates of the INPUT for supercell (S.G.: *P1*) for the structure model development. The lattice parameters are *a* = 4.928 Å, *b* = 4.928 Å, *c* = 5.030 Å, α = 80.35°, β = 99.60° and γ = 60.11°. Based on this primitive cell a 3×2×2 supercell was built.

Atom	*x*	*Y*	*Z*	S.O.F
Mn1	0.167	0.167	0	0.5
Ni1	0.167	0.167	0	0.5
Mn2	-0.167	-0.167	0	0.5
Ni2	-0.167	-0.167	0	0.5
Li1	-0.5	-0.5	0.0	0.6
Mn3	-0.5	-0.5	0.0	0.4
Li2	0.0	0.0	0.5	1.0
Li3	0.34	0.34	0.5	1.0
Li4	-0.34	-0.34	0.5	1.0
O1	0.218	-0.218	0.227	1.0
O2	-0.218	0.218	-0.227	1.0
O3	-0.424	0.067	0.223	1.0
O4	0.067	-0.428	-0.223	1.0
O5	0.424	-0.067	-0.223	1.0
O6	-0.067	0.425	0.223	1.0

Supplementary Table 7 Atomic coordinates of structure model ($Li_{44}Mn_{18}Ni_{10}O_{72}$, *P1*) after optimization with CRYSTAL17. The lattice parameters are *a* = 14.7077 Å, *b* = 9.8281 Å, *c* = 9.9263 Å, α = 80.67°, β = 99.44° and γ = 59.83°.

Atom	*x*	*y*	*z*
Li1	0.833154	0.749788	-0.000274
Li2	0.834210	0.745464	0.503517
Li3	0.833559	0.250052	-0.000407
Li4	0.833680	0.252563	0.497692
Li5	0.166770	0.750454	0.000146
Li6	0.166788	0.248908	-0.000227
Li7	0.499494	0.750013	0.000569
Li8	0.499828	0.249819	-0.000012
Li9	-0.004944	0.006427	0.245249
Li10	-0.000372	0.000792	0.747547
Li11	-0.003656	0.505299	0.246479
Li12	-0.000275	0.499319	0.748760
Li13	0.329497	0.007079	0.247461
Li14	0.337524	-0.005028	0.753369
Li15	0.331630	0.501406	0.251766
Li16	0.336444	0.494112	0.751160
Li17	0.665519	0.001507	0.249418
Li18	0.669461	-0.005897	0.752730
Li19	0.667222	0.501189	0.253557
Li20	0.669204	0.497519	0.750870

Li21	0.111020	0.172430	0.246076
Li22	0.110865	0.169348	0.755464
Li23	0.113288	0.667051	0.246529
Li24	0.111353	0.667347	0.753576
Li25	0.446299	0.165898	0.248652
Li26	0.445005	0.167877	0.750557
Li27	0.446590	0.666084	0.244472
Li28	0.446383	0.666304	0.750478
Li29	0.778746	0.172674	0.250492
Li30	0.780107	0.170799	0.751159
Li31	0.779423	0.668221	0.253774
Li32	0.781138	0.667404	0.746950
Li33	0.885919	0.828824	0.248989
Li34	0.886837	0.828317	0.749688
Li35	0.886924	0.330727	0.251009
Li36	0.885701	0.333606	0.748061
Li37	0.222215	0.829785	0.244661
Li38	0.220663	0.830310	0.755892
Li39	0.222670	0.331368	0.247913
Li40	0.219976	0.334699	0.749697
Li41	0.557157	0.828185	0.250856
Li42	0.553612	0.834527	0.752090
Li43	0.555229	0.332724	0.248072
Li44	0.553820	0.333478	0.752726
Mn1	0.167441	0.747949	0.501959
Mn2	0.166594	0.252433	0.496847
Mn3	0.500113	0.749925	0.500113
Mn4	0.499785	0.250024	0.500537
Mn5	0.056643	0.082318	-0.000762
Mn6	0.055365	0.583167	-0.000469
Mn7	0.388017	0.082030	0.000131
Mn8	0.388088	0.584985	-0.000116
Mn9	0.722346	0.083375	0.000677
Mn10	0.724801	0.081513	0.500683
Mn11	0.722380	0.583254	0.000487
Mn12	0.944451	0.916552	-0.000487
Mn13	0.942907	0.918872	0.500366
Mn14	0.944298	0.416422	-0.001179
Mn15	0.940969	0.417889	0.499620
Mn16	0.277936	0.416393	0.000136
Mn17	0.611293	0.916057	0.001018
Mn18	0.611176	0.416919	0.000526
Ni1	0.054162	0.084576	0.498094
Ni2	0.049995	0.587516	0.499579
Ni3	0.388777	0.084420	0.499717
Ni4	0.388239	0.582960	0.501192
Ni5	0.723708	0.581617	0.500723
Ni6	0.277645	0.916444	0.000138
Ni7	0.277191	0.918363	0.500357
Ni8	0.278764	0.415513	0.500127
Ni9	0.616530	0.910265	0.499843
Ni10	0.616063	0.414733	0.499762
O1	0.072712	0.891257	0.111929
O2	0.079160	0.872097	0.616200
O3	0.072528	0.391160	0.110958
O4	0.081299	0.371107	0.613913
O5	0.403697	0.891166	0.110455
O6	0.416212	0.868979	0.616243
O7	0.405849	0.392246	0.111460
O8	0.424597	0.363564	0.619951
O9	0.739840	0.890484	0.114493
O10	0.744894	0.887307	0.615546
O11	0.739888	0.390513	0.113872
O12	0.743465	0.390886	0.611291
O13	0.927590	0.108679	0.885239
O14	0.925769	0.110274	0.387976
O15	0.926885	0.609115	0.886472

O16	0.922893	0.612370	0.385314
O17	0.260112	0.105646	0.889491
O18	0.243348	0.133859	0.379733
O19	0.260184	0.608644	0.888255
O20	0.241878	0.637366	0.381055
O21	0.594349	0.108073	0.889410
O22	0.589231	0.123986	0.385829
O23	0.593818	0.608839	0.888678
O24	0.576776	0.631371	0.380579
O25	0.858574	0.034264	0.112768
O26	0.858620	0.034994	0.613629
O27	0.858616	0.533697	0.111666
O28	0.867965	0.527843	0.618591
O29	0.192441	0.033024	0.111192
O30	0.191401	0.047203	0.604588
O31	0.191507	0.534119	0.113122
O32	0.188404	0.555057	0.613367
O33	0.524534	0.033178	0.113805
O34	0.529422	0.048917	0.613724
O35	0.524825	0.534818	0.112709
O36	0.528148	0.550241	0.613642
O37	0.022942	0.786731	0.887490
O38	0.028416	0.796074	0.387191
O39	0.023524	0.285670	0.887140
O40	0.029624	0.296313	0.384158
O41	0.355025	0.789504	0.888995
O42	0.360841	0.800852	0.390610
O43	0.355498	0.286395	0.887098
O44	0.372326	0.288715	0.382135
O45	0.689638	0.786800	0.887912
O46	0.694433	0.778867	0.390839
O47	0.689761	0.286899	0.887383
O48	0.693885	0.282649	0.386429
O49	0.143871	0.963558	0.888104
O50	0.139454	0.952761	0.392112
O51	0.141730	0.464719	0.887199
O52	0.137913	0.450544	0.385761
O53	0.475188	0.965237	0.887125
O54	0.477668	0.945169	0.386236
O55	0.474533	0.467311	0.886723
O56	0.477894	0.445368	0.386933
O57	0.808429	0.965935	0.887674
O58	0.809153	0.965241	0.387165
O59	0.808192	0.465720	0.886874
O60	0.811744	0.461479	0.385037
O61	0.977979	0.211941	0.112454
O62	0.977982	0.209813	0.609229
O63	0.977058	0.713082	0.112741
O64	0.973702	0.716902	0.613296
O65	0.310460	0.212426	0.113015
O66	0.294563	0.210339	0.617713
O67	0.309732	0.715566	0.111895
O68	0.294065	0.713632	0.618235
O69	0.643479	0.213172	0.112524
O70	0.639486	0.204230	0.613290
O71	0.643825	0.712636	0.113110
O72	0.633203	0.708148	0.614206
